# Cardiovascular risk of metabolically healthy obesity in two european populations: Prevention potential from a metabolomic study

**DOI:** 10.1186/s12933-023-01815-6

**Published:** 2023-04-07

**Authors:** Dongmei Wei, Vannina González-Marrachelli, Jesus D Melgarejo, Chia-Te Liao, Angie Hu, Stefan Janssens, Peter Verhamme, Lucas Van Aelst, Thomas Vanassche, Josep Redon, Maria Tellez-Plaza, Juan C Martin-Escudero, Daniel Monleon, Zhen-Yu Zhang

**Affiliations:** 1 Studies Coordinating Centre, Research Unit Hypertension and Cardiovascular Epidemiology, Department of Cardiovascular Sciences, KU Leuven, University of Leuven, Campus Sint Rafaël, Kapucijnenvoer 7, block h, Box 7001, Leuven, BE- 3000 Belgium; 2 Department of Physiology, Faculty of Medicine, University of Valencia, Valencia, Spain; 3 Institute for Biomedical Research, Hospital Clinic of Valencia (INCLIVA), Valencia, Spain; 4 Faculty of Medicine and Dentistry, University of Alberta, Edmonton, Canada; 5 Division of Cardiology, University Hospitals Leuven, Leuven, Belgium; 6 Department of Cardiovascular Sciences, University of Leuven, Leuven, Belgium; 7 Department of Preventive Medicine and Microbiology, Universidad Autónoma de Madrid, Madrid, Spain; 8 Integrative Epidemiology Group, Department of Chronic Diseases Epidemiology, National Center for Epidemiology, Carlos III Health Institute, Madrid, Spain; 9 Department of Internal Medicine, Hospital Universitario Rio Hortega, University of Valladolid, Valladolid, Spain; 10 Department of Pathology, University of Valencia, Valencia, Spain

**Keywords:** Cardiovascular risk, Diabetes, Obesity, Metabolically healthy obesity, Metabolomics

## Abstract

**Background:**

A new definition of metabolically healthy obesity (MHO) has recently been proposed to stratify the heterogeneous mortality risk of obesity. Metabolomic profiling provides clues to metabolic alterations beyond clinical definition. We aimed to evaluate the association between MHO and cardiovascular events and assess its metabolomic pattern.

**Methods:**

This prospective study included Europeans from two population-based studies, the FLEMENGHO and the Hortega study. A total of 2339 participants with follow-up were analyzed, including 2218 with metabolomic profiling. Metabolic health was developed from the third National Health and Nutrition Examination Survey and the UK biobank cohorts and defined as systolic blood pressure < 130 mmHg, no antihypertensive drugs, waist-to-hip ratio < 0.95 for women or 1.03 for men, and the absence of diabetes. BMI categories included normal weight, overweight, and obesity (BMI < 25, 25–30, ≥ 30 kg/m^2^). Participants were classified into six subgroups according to BMI category and metabolic healthy status. Outcomes were fatal and nonfatal composited cardiovascular events.

**Results:**

Of 2339 participants, the mean age was 51 years, 1161 (49.6%) were women, 434 (18.6%) had obesity, 117 (5.0%) were classified as MHO, and both cohorts had similar characteristics. Over a median of 9.2-year (3.7–13.0) follow-up, 245 cardiovascular events occurred. Compared to those with metabolically healthy normal weight, individuals with metabolic unhealthy status had a higher risk of cardiovascular events, regardless of BMI category (adjusted HR: 3.30 [95% CI: 1.73–6.28] for normal weight, 2.50 [95% CI: 1.34–4.66] for overweight, and 3.42 [95% CI: 1.81–6.44] for obesity), whereas those with MHO were not at increased risk of cardiovascular events (HR: 1.11 [95% CI: 0.36–3.45]). Factor analysis identified a metabolomic factor mainly associated with glucose regulation, which was associated with cardiovascular events (HR: 1.22 [95% CI: 1.10–1.36]). Individuals with MHO tended to present a higher metabolomic factor score than those with metabolically healthy normal weight (0.175 vs. -0.057, P = 0.019), and the score was comparable to metabolically unhealthy obesity (0.175 vs. -0.080, P = 0.91).

**Conclusions:**

Individuals with MHO may not present higher short-term cardiovascular risk but tend to have a metabolomic pattern associated with higher cardiovascular risk, emphasizing a need for early intervention.

**Supplementary information:**

The online version contains supplementary material available at 10.1186/s12933-023-01815-6.

## Introduction

Obesity is closely associated with cardiometabolic disorders. However, people with obesity are at heterogeneous risk of cardiometabolic diseases. Metabolically healthy obesity (MHO) refers to the concept of obesity without cardiometabolic disorders [1]. It is still a subject of debate whether individuals with MHO are truly at equivalent cardiovascular risk compared to metabolically healthy normal weight. The inconsistent evidence can be partially attributed to a lack of consensus on metabolic health that causes 30 varying definitions applied in previous studies [2]. Although previous standards of MHO are mainly based on the absence of metabolic syndromes, the criteria and cutoff values used varied considerably [2].  A large prospective study on the third National Health and Nutrition Examination Survey and the UK biobank cohort systematically evaluated various metabolic risk factors and proposed a new definition of metabolic health derived from mortality risk [3]. This new definition based on systolic blood pressure, use of antihypertensives, waist-to-hip ratio, and self-reported diabetes could stratify mortality risk for individuals with and without obesity [3]. Furthermore, metabolic health status evolved over time for most people, including individuals with normal weight [4, 5]. One-time classification might be inadequate, whereas complicated criteria might burden the dynamic evaluations. Therefore, this simple definition may be more feasible in clinical contexts. It is unclear, however, whether the new definition can be generalized to cardiovascular events in the general population.

Comprehensive metabolomic profiling may provide molecular pathophysiological insight into the heterogeneity of obesity. Previous cross-sectional metabolomics studies have reported clusters of metabolites associated with MHO [6–9]. Nonetheless, simultaneous investigation of the association between obese phenotypes, cardiovascular events, and a large scale of metabolites could further underpin the metabolic pathways significant to obesity. Therefore, our primary hypothesis was that circulating metabolites associated with cardiovascular risk would identify subtle metabolism disorders for individuals with MHO, prior to evident cardiovascular risk. We first studied the cardiovascular risk for individuals with MHO to examine whether the new definition can be generalized for risk stratification of cardiovascular events in 2339 Europeans. Subsequently, with metabolomic profiling in 2218 participants, we identified metabolites distinct between different phenotypes classified by the new definition and assessed its association with cardiovascular events.

## Methods

This study was designed as prospective multicenter study. Study participants were from two prospective population-based studies, the Flemish Study on Environment, Genes and Health Outcomes (FLEMENGHO) study and the Hortega Study. The FLEMENGHO study and the Hortega study were approved by the Institutional Review Board at the University of Leuven and the University Hospital Rio Hortega (UHRH), respectively. All participants provided written informed consent. This study followed the Strengthening the Reporting of Observational Studies in Epidemiology (STROBE) reporting guideline for cohort studies.

### Study participants

The FLEMENGHO study enrolled participants from the Flemish region in Belgium from 1985 to 2004 with repetitive follow-ups. From 2005 to 2010, 1331 participants from the FLEMENGHO study provided plasma samples for metabolomic profiling, and all of them were followed up. The Hortega Study is a prospective study that sought to investigate traditional and non-traditional risk factors for chronic diseases in adult beneficiaries from the public health system of UHRH in Spain. Study protocols, inclusion criterions, and baseline characteristics were described elsewhere [10, 11]. Between 2001 and 2003, 1502 individuals from the Hortega Study underwent baseline examinations [11]. Participants from the Hortega study were excluded due to the missing information on body mass index (BMI, n = 58), waist-to-hip ratio (n = 57), blood glucose (n = 180), prevalent cardiovascular event (n = 112), and loses to follow-up (n = 87). Thus, 1008 participants from the Hortega study were included, with subset of 887 having available metabolomic data. Overall, the investigation on the association of metabolic phenotypes with cardiovascular events including both cohorts was performed on a total of 2339 participants, and the metabolomic analysis was conducted in 2218 participants.

### Classifications of metabolic health and obesity

Metabolic health was defined by the new definition as systolic blood pressure < 130 mmHg, no antihypertensive drugs, waist-to-hip ratio < 0.95 for women or 1.03 for men, and the absence of diabetes [3]. Meanwhile, as a comparison, metabolic health was defined by the absence of metabolic syndrome (MetS) according to the 2009 Joint Interim Statement [12]. With the MetS definition, participants were considered metabolic healthy if having ≤ 2 of the following disorders: systolic/diastolic blood pressure ≥ 130/85 mmHg or use of antihypertensive medication; triglyceride level ≥ 1.70 mmol/L or use of lipid-lowering medication; high-density lipoprotein cholesterol level < 1 mmol/L in men or < 1.30 mmol/L in women; and fasting glucose level ≥ 5.60 mmol/L or non-fasting blood glucose ≥ 7.80 mmol/L or history of diabetes; waist circumference ≥ 94 cm in men or ≥ 80 cm in women. BMI categories included normal weight (BMI < 25 kg/m^2^), overweight (25 ≤ BMI < 30 kg/m^2^), and obesity (≥ 30 kg/m^2^). Participants were classified into six subgroups by their metabolic health status and BMI categories: metabolically healthy normal weight, metabolically healthy overweight, MHO, metabolically unhealthy normal weight, metabolically unhealthy overweight, and metabolically unhealthy obesity.

### Metabolomic profiling

The venous blood samples were obtained from the FLEMENGHO study after at least 8 h of fasting. Metabolomic data from the Hortega study was corrected by fasting hours and last triglyceride levels before sampling to adjust for the effect of diets. Metabolic profiling was performed with H1 nuclear magnetic resonance (NMR) spectrometry at the INCLIVA Molecular and Metabolomics Image Lab, Valencia, Spain [13]. Sample preparation and detailed methods were previously described and provided in the supplementary method [14]. Briefly, 1 H NMR spectra was acquired by Bruker Avance 600 spectrometer operating at 600.13 MHz with a 5-mm 1 H/13 C/15 N TXI probe. Resonances in spectral regions were assigned according to the Human Metabolome Database and selected two-dimensional NMR spectra [15]. The metabolite relative abundances were calculated by spectral region area integration and normalized to the total aliphatic spectral area. Topspin 3 1.3 (Bruker Biospin GmbH, Karlsruhe, Germany) and MATLAB® (MathWorks Inc., version 4 2013a) were used for spectra processing and analysis. NMR spectroscopy is a high-throughput, quantitative technique to measure multiple metabolites simultaneously, and it shows an advantage in profiling metabolites that are not fully investigated but potentially related to the outcomes of interest [16, 17]. The crowded spectra produced by NMR spectrometry with a fast speed may not be always deconvoluted into single metabolites because magnetically equivalent hydrogen atom that produces a characteristic signal in a region can be found in several metabolites [16, 18]. Therefore, two metabolites were jointly reported as composite metabolites when their peaks contributed to a relevant spectral region. Nonetheless, NMR-measured metabolomics is noteworthy and can provide new insights into disease mechanisms, and given the cost-effectiveness of NMR, it is suitable for large epidemiological investigations [19]. The reproducibility of NMR spectrometry was routinely examined, and the variation was less than 5%.

### Assessments of Cardiovascular Outcomes

Primary outcomes consisted of fatal and nonfatal cardiovascular events. cardiovascular event was defined as myocardial infarction, acute coronary syndrome, new-onset angina pectoris, ischemic cardiomyopathy, coronary revascularization, heart failure, new-onset atrial fibrillation, life-threatening arrhythmias, pulmonary heart disease, sudden death, aortic aneurysm, peripheral artery disease, peripheral artery revascularization, stroke, and transient ischemic attack. Individuals from the FLEMENGHO study and the Hortega study were followed up until December 31, 2016, and November 30, 2015, respectively. Incident cardiovascular events were adjudicated from several information sources including study visits (the Hortega study) and/or telephone call (the FLEMENGHO study), hospital discharge lists, medical records, and death certificates [10, 11]. The incident cardiovascular events were adjudicated using the ICD-10 (I20-I25, I48-I50, I63, I71.4, I73.9, Z98.61).

### Other measurements

BMI, waist circumference, and hip circumference were measured using standard protocols. Blood pressure was calculated as the mean of five consecutive auscultatory readings with a mercury sphygmomanometer. Hypertension was defined as an office blood pressure of ≥ 140 mmHg (systolic) or ≥ 90 mmHg (diastolic), or the use of antihypertensive drugs. Diabetes mellitus was defined as fasting blood glucose of ≥ 7.00 mmol/L or random blood glucose ≥ 11.1 mmol/L or treatment with antidiabetic drugs in the FLEMENGHO study. The Hortega study defined diabetes as fasting blood glucose > 7.00 mmol/L or HbA1c ≥ 6.5% or a diabetes history or with antidiabetic drugs. Biochemistry test included total cholesterol, low-density lipoprotein cholesterol, high-density lipoprotein cholesterol, triglyceride, blood glucose, and serum creatinine by using automated methods in certified laboratories [11, 20].

### Statistical analyses

Statistical significance was a two-sided P value of 0.05. Data analyses were performed with SAS software, version 9.4 (SAS Institute) or Python, version 3.9. Categorical variables were reported as frequencies with percentages and continuous variables were reported as means with standard deviations (SDs) or medians with interquartile ranges (IQRs). Baseline characteristics were compared using Chi-squared test for categorical variables, t test or Wilcoxon rank sum test for continuous variables as appropriate. Cox proportional regression models were used to examine the association of baseline metabolic health phenotypes with cardiovascular outcomes. Covariates used in the Cox regression analysis included sex, age, total physical activity, smoking, and alcohol consumption at baseline. When merging two cohorts, the cohort indicator and the interaction term of cohorts were additionally included in preliminary analysis. Since the interaction term was not statistically significant, subsequent analysis did adjust for a cohort indicator but did not include the interaction term. Sensitivity analyses of the association between metabolic health phenotypes and cardiovascular events were performed after excluding individuals with underweight (BMI < 18.5 kg/m^2^) and severe obesity (BMI > 40 kg/m^2^) or on lipid-lowering medications.

The metabolomic characterization was explored in the following steps. First, the metabolite data were normalized and scaled by subtracting the mean of each metabolite and dividing by its standard deviation, and each metabolite would have a mean of zero and a standard deviation of one. This transformation was performed within each cohort due to the batch effect and the potential influence caused by cohort characteristics. After this data conversion, the metabolites data from the two cohorts were merged into a whole dataset. The transformed data were visualized by histogram plots (Figures S1-3 in the supplementary material). Second, the levels of 44 metabolites in the whole dataset at baseline were compared between six metabolic subgroups. After scaling and normalization, the metabolites were normally distributed in the whole dataset, as shown in Figures S1-3 in the supplementary material. When performing the group-wise comparison, these metabolites did not always statistically fit the normal distribution in each subgroup and consistently pass the normality test required for parametric tests. Therefore, the Kruskal-Wallis test was used to obtain more robust results. Metabolites were considered significant if their P values was less than 0.05 after the Bonferroni correction. Next, the significant metabolites were combined by using factor analysis. Exploratory factor analysis was used to uncover the underlying structure of significant metabolites correlated with each other [21]. Exploratory factor analysis reduced the data dimensions by identifying fewer latent factors that can explain the variation in significant metabolites. The Kaiser-Meyer-Olkin (KMO) test was performed to determine whether the data was suitable for factor analysis. Factors with an eigenvalue greater than 2 were retained. The strength of the correlation between the identified factors and metabolites can be interpreted by standardized factor loadings. A factor loading was in the range of zero to one, and a loading close to one indicates a strong correlation. Subsequently, the association between constructed factors, major relevant metabolites, and cardiovascular events were evaluated by multivariate Cox regression models with adjustment of covariates and the cohort indicator. Same as before, since the interaction coefficient by cohort was not statistically significant, we only retained the main effect cohort adjustment in subsequent analysis. The level of cardiovascular events-associated factors was compared across six metabolic subgroups.

## Results

### Participant characteristics

Of 2339 participants, the mean (SD) age of participants was 51.2 (16.9) years and 1161 (49.6%) were women. BMI was 26.4 (4.4) kg/m^2^, and 434 (18.6%) participants had obesity. According to the new definition, 117 (5.0%) participants with obesity were metabolically healthy and 317 (13.6%) were metabolically unhealthy. With the Mets-based definition, 251 (10.7%) had MHO and 183 (7.8%) had metabolically unhealthy obesity. Baseline characteristics of participants in subgroups classified by the new definition are shown in Table 1. As expected, compared with individuals with metabolically unhealthy obesity, participants with MHO tended to be younger and were less likely to have history of cardiovascular disease, diabetes, hypertension, receive antihypertensive and lipid-lowering agents, and tended to have lower blood pressure, waist circumference, and blood glucose (P ≤ 0.002). Few participants classified as metabolically healthy, such as MHO, were diagnosed with hypertension due to diastolic blood pressure of ≥ 90 mmHg. Physical activities in individuals with MHO were higher, even though not reaching significance. Most characteristics from the FLEMENGHO and the Hortega study were similar, such as age (51.1 vs. 51.3 years) and female percentage (50.3% vs. 48.8%; Table S1 in the supplementary material).

**Table 1:** Baseline Characteristics of Participants Across Metabolic Phenotypes Classified by the New Definition

Characteristics	MHNW (n = 617)	MHOW (n = 384)	MHO (n = 117)	MUHNW (n = 346)	MUOW (n = 558)	MUO (n = 317)
Number with characteristic (%)						
Female	299 (48.5) †	202 (52.6)	71 (60.7)	184 (53.2)	269 (48.2)	136 (42.9) *
Current Smoking	182 (29.5)	105 (27.3)	25 (21.4)	63 (18.2)	78 (14.0)	31 (9.8) *
Current alcohol assumption	400 (64.8)	263 (68.5)	66 (56.4)	217 (62.7)	334 (59.9)	177 (55.8)
History of CVD	6 (1.0)	6 (1.6)	2 (1.7)	26 (7.5)	52 (9.3)	20 (6.3)
Diabetes mellitus	0 (0.0)	0 (0.0)	0 (0.0)	16 (4.6)	58 (10.4)	42 (13.3) *
Hypertension	5 (0.8)	13 (3.4)	8 (6.8)	237 (68.5)	138 (24.7)	257 (81.1) *
Treatment of hypertension	0 (0.0) †	0 (0.0)	0 (0.0)	109 (31.5)	217 (38.9)	155 (48.9) *
lipid-lowering drugs	15 (2.4) †	23 (6.0)	9 (7.7)	50 (14.5)	103 (18.5)	52 (16.4) *
Mean or median of characteristic						
Age, years	39.0 ± 12.8 †	45.6 ± 13.7	45.7 ± 14.0	57.2 ± 16.5	60.7 ± 14.6	60.6 ± 13.7 *
Body mass index, kg/m^2^	22.3 ± 1.8 †	27.0 ± 1.4	32.6 ± 2.6	22.9 ± 1.7	27.4 ± 1.4	33.4 ± 3.3 *
Waist-to-hip ratio	0.81 ± 0.07 †	0.88 ± 0.08	0.90 ± 0.08	0.86 ± 0.07	0.91 ± 0.08	0.94 ± 0.09 *
Waist circumference, cm	77.8 ± 8.0 †	91.0 ± 7.6	103.1 ± 9.4	83.6 ± 8.2	94.8 ± 7.6	106.6 ± 9.8 *
Hip circumference, cm	96.3 ± 5.6 †	103.9 ± 4.6	114.4 ± 7.9	97.2 ± 5.1	104.2 ± 6.3	113.5 ± 8.4
Physical activity, kcal/day	1341.2 (800.7–1954.3)	1499.6 (977.1–2085.8)	1566.1 (977.1–2103.9)	1463.3 (1082.9–2007.2)	1498.3 (1036.0–2036.3)	1484.6 (977.1–2152.5)
Systolic blood pressure, mmHg	114.4 ± 8.9 †	117.8 ± 8.4	119.9 ± 7.1	142.0 ± 15.8	142.9 ± 16.5	143.4 ± 16.2 *
Diastolic blood pressure, mmHg	73.7 ± 7.8 †	76.9 ± 7.6	80.4 ± 6.5	82.7 ± 10.0	84.3 ± 10.2	86.4 ± 9.2 *
Total cholesterol, mmol/L	5.02 ± 0.90 †	5.41 ± 0.95	5.34 ± 1.04	5.30 ± 0.95	5.45 ± 0.99	5.48 ± 1.07
LDL-cholesterol, mmol/L	3.03 ± 0.83 †	3.37 ± 0.86	3.40 ± 0.93	3.24 ± 0.85	3.40 ± 0.89	3.38 ± 0.95
HDL-cholesterol, mmol/L	1.57 ± 0.38 †	1.36 ± 0.36	1.25 ± 0.27	1.54 ± 0.41	1.36 ± 0.34	1.30 ± 0.32
Triglycerides, mmol/L	0.79 (0.56–1.08) †	1.15 (0.80–1.74)	1.31 (0.95–1.84)	0.95 (0.70–1.39)	1.30 (0.93–1.81)	1.50 (1.03–2.19)
Blood glucose, mmol/L	4.66 (4.33–5.00) †	4.77 (4.44–5.05)	4.83 (4.39–5.05)	4.66 (4.33–5.00)	4.94 (4.61–5.28)	5.00 (4.61–5.50) *
Serum creatinine, mg/dL	0.80 (0.70–0.92)	0.90 (0.76–1.00)	0.85 (0.72–0.96)	0.85 (0.72–1.00)	0.92 (0.80–1.04)	0.90 (0.75–1.02) *
eGFR, ml/min/1.73m^2^	102.3 ± 20.6	89.7 ± 19.4	92.1 ± 20.4	85.1 ± 19.2	78.9 ± 19.5	81.3 ± 18.8 *

Abbreviation: CVD, cardiovascular disease; eGFR, estimated glomerular filtration rate; MHNW, metabolically healthy normal weight; MHOW, metabolically healthy overweight; MHO, metabolically healthy obesity; MUHNW, metabolically unhealthy normal weight; MUHOW, metabolically unhealthy overweight; MUHO, metabolically unhealthy obesity. *P value < 0.05 for the comparison between MHO and MUHO. †P value < 0.05 for the comparison between MHO and MHNW.

### Association of metabolic phenotypes with Cardiovascular Outcomes

**Fig. 1:**
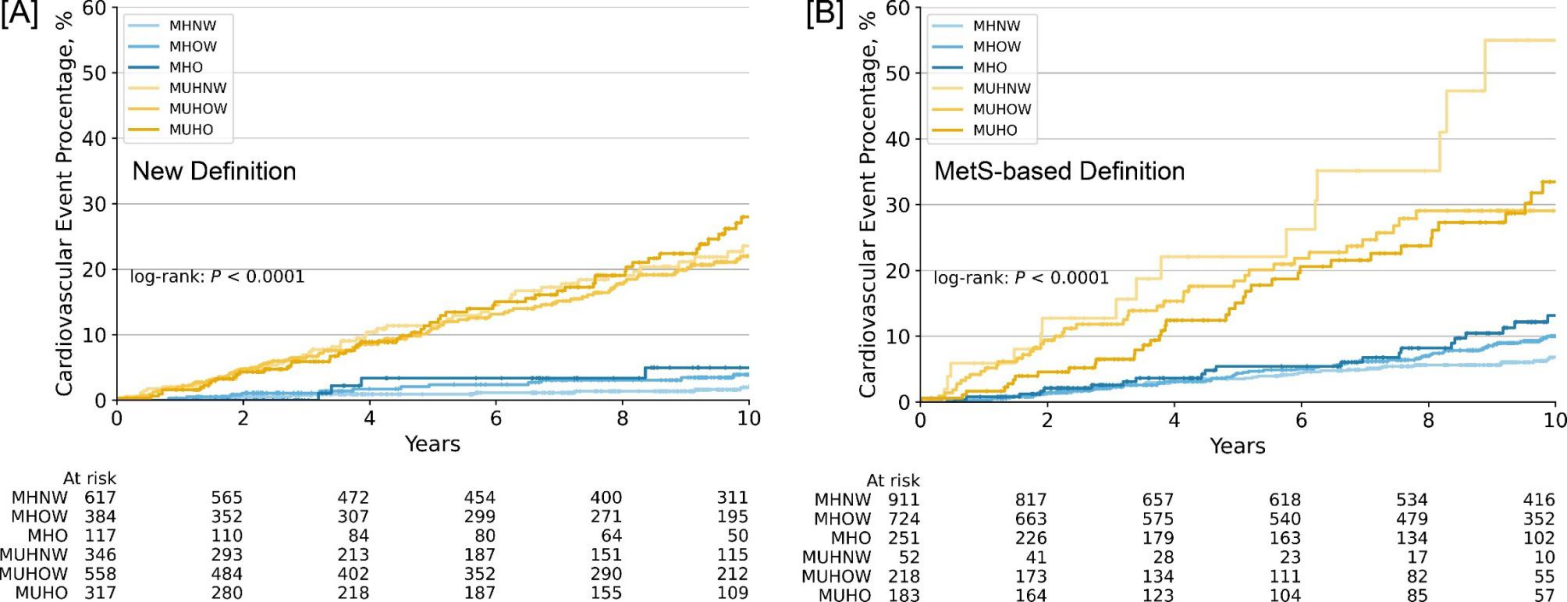
Cumulative Risk of Cardiovascular Events in Subgroups of Metabolic Healthy Status and Body Mass Index Category. Metabolic health was defined by the new definition (**A**) and the prior definition based on the absence of metabolic syndromes (Mets definition, **B**). MHNW, metabolically healthy normal weight; MHOW, metabolically healthy overweight; MHO, metabolically healthy obesity; MUHNW, metabolically unhealthy normal weight; MUHOW, metabolically unhealthy overweight; MUHO, metabolically unhealthy obesity

Over a median of 9.2-year [(IQR) 3.7–13.0] follow-up, 245 cardiovascular events occurred, 111 from the FLEMENGHO study and 134 from the Hortega study. Compared with individuals with metabolically healthy normal weight, individuals classified as metabolic unhealthy by the new definition had an increased risk of cardiovascular events, irrespective of BMI category (Fig. 1; Table 2). However, the adjusted risk of cardiovascular events for participants with MHO was not significantly increased (hazard ratio [HR]: 1.11; 95% CI: 0.36–3.45 after adjusting for covariates and the cohort indicator; Table 2). These associations were consistently observed in both study participants (Table 2). Sensitivity analyses further confirmed that MHO was not associated with cardiovascular events, whereas the cardiovascular risk of participants with metabolic unhealthy status was significantly increased independent of the BMI category (Table S2 in the supplementary material).

**Table 2:** Association between Metabolic Phenotypes and Cardiovascular Events

	All (N = 2339)			Flemengho (N = 1331)			Hortega (N = 1008)	
	HR (95% CI) *	*P*		HR (95% CI)	*P*		HR (95% CI)	*P*
New definition								
MHNW	1 (reference)			1 (reference)			1 (reference)	
MHOW	1.22 (0.57–2.62)	0.60		1.47 (0.42–5.10)	0.54		0.98 (0.37–2.63)	0.97
MHO	1.11 (0.36–3.45)	0.86		2.34 (0.43–12.82)	0.33		0.65 (0.14–3.06)	0.58
MUHNW	3.30 (1.73–6.28)	0.0003		3.18 (1.06–9.51)	0.038		3.21 (1.44–7.14)	0.004
MUHOW	2.50 (1.34–4.66)	0.004		3.37 (1.17–9.74)	0.025		1.76 (0.81–3.83)	0.151
MUHO	3.42 (1.81–6.44)	0.0001		3.04 (1.01–9.13)	0.047		3.39 (1.56–7.37)	0.002
**MetS-Based definition**								
MHNW	1 (reference)			1 (reference)			1 (reference)	
MHOW	0.83 (0.57–1.21)	0.34		1.25 (0.69–2.25)	0.47		0.63 (0.39–1.01)	0.055
MHO	1.11 (0.69–1.77)	0.68		0.75 (0.28–2.01)	0.56		1.17 (0.67–2.02)	0.59
MUHNW	2.09 (1.18–3.72)	0.012		2.06 (0.92–4.58)	0.078		2.06 (0.84–5.04)	0.12
MUHOW	1.57 (1.04–2.37)	0.033		1.62 (0.89–2.96)	0.11		1.44 (0.79–2.63)	0.23
MUHO	1.83 (1.21–2.79)	0.004		1.87 (0.99–3.55)	0.055		1.74 (0.99–3.05)	0.054

All HRs were adjusted for sex, age, total physical activity, smoking, and alcohol consumption. * HRs were additionally adjusted for the cohort indicator

Abbreviation: MHNW, metabolically healthy normal weight; MHOW, metabolically healthy overweight; MHO, metabolically healthy obesity; MUHNW, metabolically unhealthy normal weight; MUHOW, metabolically unhealthy overweight; MUHO, metabolically unhealthy obesity; HR, hazard ratio.

With the MetS-based definition, an increased risk of cardiovascular events was observed for individuals classified as metabolically unhealthy as well (Fig. 1). Of note, individuals with metabolically healthy normal weight or MHO classified by the MetS-based definition had higher cardiovascular risk than those with same status classified by the new definition. The significant association between metabolic unhealthy status and cardiovascular events was observed in 2339 participants, independent of their BMI category (Table 2). However, this association was unable to be separately observed in the FLEMENGHO or Hortega cohort.

### Metabolomic factors

A total of 44 metabolites were detected, covering lipids, carbohydrates, amino acids, and organic acids, as shown in Figure S4 in the supplementary material. There were 38 metabolites that significantly differed in different metabolic subgroups. There were 28 metabolites significantly different between MHO and metabolically healthy normal weight, as shown in Figure S5 in the supplemental material. Compared MHO to metabolically unhealthy obesity, 12 metabolites were significantly different. Further factor analysis constructed five components that explained 73.2% of the variance in metabolites (Table S3 in the supplementary material). The first factor represented 2-aminobutyrate, 4-aminobutyrate, alanine, acetate, branched-chain amino acids (valine, leucine, isoleucine), and -CH = CH, while the second factor mainly consisted of glucose + glutamine, glucose + 2-aminobutyrate, glucose + 2-phosphoglycerate, glucose, unknown molecule, and glycine (Figure S6 in the supplementary material). The correlation of clinically measured glucose with factor 2 and glucose-related metabolites (glucose, glucose + glutamine, etc.) varied from 0.01 to 0.37, as shown in Figure S7 in the supplementary material. The third factor summarized phenylalanine, glutamine, glutamate, FA = CH-CH2-CH2 = + citrate + aspartate, and the fourth factor primarily comprised HDL3, tyrosine, and valerate. The fifth factor contained valine, FA a-CH2, and creatinine.

**Fig. 2:**
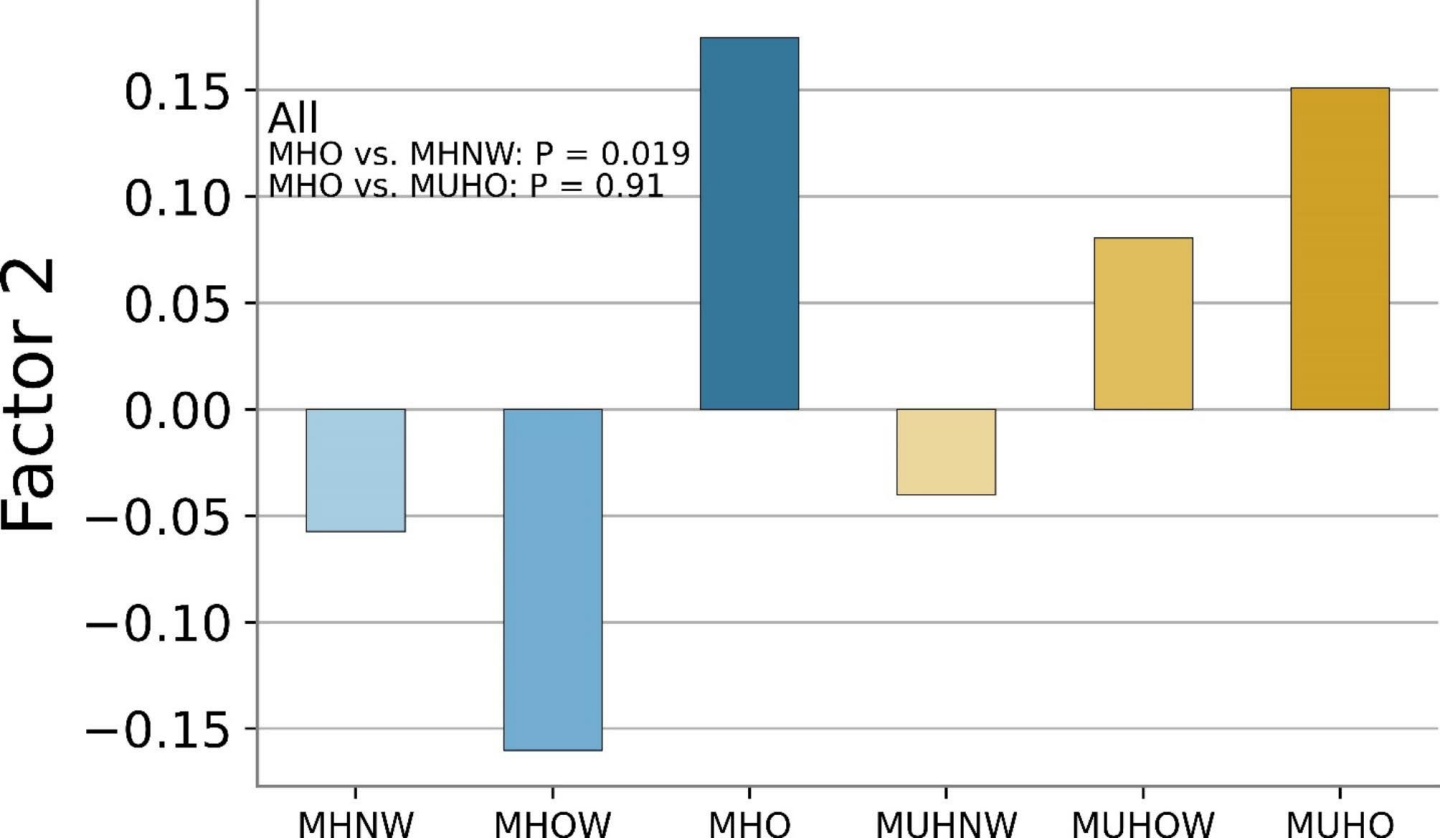
Cardiovascular Risk-associated Metabolomic Factor in Subgroups of Metabolic Healthy Status and Body Mass Index Category. MHNW, metabolically healthy normal weight; MHOW, metabolically healthy overweight; MHO, metabolically healthy obesity; MUHNW, metabolically unhealthy normal weight; MUHOW, metabolically unhealthy overweight; MUHO, metabolically unhealthy obesity

Survival analysis suggested that the second factor mainly related to glucose metabolism showed a positive association with cardiovascular events (HR: 1.24; 95% CI: 1.09–1.40; Table 3), whereas other factors did not (Table S4 in the supplementary material). To examine whether the association between MHO and cardiovascular events was mediated by the metabolomics pattern, we estimated the direct effect of MHO on cardiovascular events by including the metabolomics pattern in the COX regression model with adjustment of sex, age, total physical activity, smoking, alcohol consumption, and the cohort indicator. The association between MHO and cardiovascular events remained insignificant (HR: 1.21 [0.38–3.81], P = 0.75), while the metabolic pattern was still associated with an increased risk of cardiovascular events (HR: 1.25 [1.12–1.40], P < 0.0001). The major metabolites in factor 2, including glucose + glutamine, glucose + 2-aminobutyrate, glucose + 2-phosphoglycerate, and glucose, were also associated with cardiovascular events. Individuals classified as metabolic unhealth had higher scores of the second factor, compared to those with metabolic health (0.064 vs. -0.069, P < 0.0001). However, the second factor score in individuals with MHO was significantly higher than the score in those with metabolically healthy normal weight (0.175 vs. -0.057, P = 0.019). For participants with obesity, the second factor scores between metabolic unhealth and health were comparable (0.175 vs. -0.080, P = 0.91, Fig. 2). This was also observed in the FLEMENGHO and Hortega study (Figure S8 in the supplementary material).

**Table 3:** Association between Metabolites and Cardiovascular Events

	All (N = 2218)			Flemengho (N = 1331)			Hortega (N = 887)	
	HR (95% CI) *	*P*		HR (95% CI)	*P*		HR (95% CI)	*P*
Factor 2	1.22 (1.10–1.36)	0.0002		1.20 (1.05–1.37)	0.006		1.37 (1.24–1.04)	0.017
Major represented metabolites								
Glucose + glutamine	1.24 (1.09–1.40)	0.0007		1.23 (1.04–1.45)	0.016		1.45 (1.23–1.02)	0.031
Glucose + 2-aminobutyrate	1.20 (1.06–1.36)	0.004		1.12 (0.94–1.33)	0.22		1.33 (1.25–1.05)	0.013
Glucose + 2-phosphoglycerate	1.19 (1.06–1.35)	0.005		1.15 (0.98–1.35)	0.079		1.35 (1.24–1.02)	0.030
Glucose	1.20 (1.06–1.36)	0.004		1.12 (0.93–1.34)	0.24		1.34 (1.23–1.04)	0.018
Unknown molecule	1.24 (1.09–1.42)	0.001		1.22 (1.01–1.46)	0.035		1.46 (1.27–1.05)	0.012
Glycine	1.04 (0.92–1.18)	0.51		1.03 (0.86–1.24)	0.75		1.24 (1.11–0.92)	0.27

HRs were calculated for per 1-unit Z score increment in metabolites and adjusted for sex, age, total physical activity, smoking, and alcohol consumption. * HRs were additionally adjusted for the cohort indicator. HR, hazard ratio.

## Discussion

In this population-based prospective study, our findings suggested that the new definition of metabolically healthy obesity could be extended to stratify cardiovascular risk for individuals with obesity. Individuals classified as MHO were not at increased risk of cardiovascular events, whereas those with metabolically unhealthy obesity did present higher risk. In participants with metabolomic profiling, we identified a circulating metabolomic factor associated with cardiovascular events, independent of cohort indicator, sex, age, current smoking, alcohol assumption, and physical activity. The metabolomic factor mainly consisted of glucose, glutamine, phosphoglycerate that were found to be individually associated with cardiovascular events as well. The metabolomic factor score was higher in individuals with metabolic unhealth. However, individuals with obesity consistently had higher proportion of the unfavorable metabolomic factor than those with normal weight or overweight, irrespective of metabolic healthy status. It is tempting to speculate that the cardiovascular risk of people with obesity can be stratified using the simple classification of metabolic health. However, our metabolomic profiling suggested that MHO classified by the new definition was not completely healthy, in terms of more evident unfavorable metabolic alterations in individuals with MHO than those with metabolic healthy normal weight. Meanwhile, the unfavorable metabolic alterations underlying the seemingly healthy phenotype of MHO could provide insights into the heterogeneity of obesity.

To our knowledge, no recent studies have evaluated whether the new definition can be extended from stratifying all-cause mortality and cardiovascular mortality to the risk assessment of fatal and nonfatal cardiovascular events, which may pave the way for achieving a uniform definition of metabolic health. This improved the interpretation of metabolic alterations and outcomes behind different obesity phenotypes. As an alternative approach to using the criteria for metabolic syndrome, Zembic et al. resolved this issue by deriving a new definition according to mortality risk and validating it in large cohorts [3]. The new definition of metabolic health was based on the systematic investigation of anthropometric and metabolic factors and their associations with all-cause mortality and cardiovascular mortality [3]. Compared with prior definitions based on the absence of MetS, the performance of the new definition was the most robust [3]. We also observed more consistent association between obese phenotypes and cardiovascular events for the new definition. This new definition underscored waist-to-hip ratio. Although both waist-to-hip ratio and waist circumference are indicators of abdominal obesity, waist-to-hip ratio is less correlated with BMI than waist circumference and is more indicative of fat distribution. The lower collinearity might explain why the addition of waist-to-hip ratio to BMI was more likely to improve the C statistics and risk reclassification for cardiovascular diseases and mortality than the addition of waist circumference [22, 23]. More importantly, metabolic health may be a transient status in people with obesity. The Whitehall II cohort study reported that around 50% of individuals who had healthy obesity at baseline converted to unhealthy obesity after 20 years of follow-up [24]. In a period of 5–10 years, 30–65% of people with MHO at baseline converted to unhealthy status [5, 25, 26]. The transient nature of metabolic health requires dynamic evaluations to reasonably assess cardiovascular risk and mortality. By including these easily obtained non-laboratory markers, this new definition can be feasibly implemented in clinical settings and research.

The unfavorable metabolomic factor existed in some individuals with MHO included metabolites associated with glycogenesis and insulin regulations. Previous studies have shown that insulin resistance is an independent risk factor for mortality, even in the absence of diabetes [27]. Long-term exposure to metabolic disorders would eventually increase the risk for mortality and cardiovascular diseases. This indicated that the phenotype of MHO is not completely healthy and requires active intervention, such as adopting healthy lifestyle. The metabolomic factor associated with cardiovascular outcomes provided molecular insights for metabolic alterations of obesity that is relevant to future studies on mechanisms, biomarkers, and potential treatment targets. Phosphoglycerate is a glycolysis-related intermediate and is suggested to be associated with an increased risk of heart failure [28]. Consisting with previous studies, glutamine is negatively associated with diabetes risk and was found lower in participants with MHO in this study [29–31]. Other metabolites that significantly differed across metabolic subgroups were reported to be associated with cardiovascular risk, although they did not show independent association in our study. Acetate, obtained either from diets or produced by gut microbes based on indigestible foods, can beneficially influence glucose hemostasis and insulin sensitivity [32]. Valerate, a short chain fatty acid, was elevated in individuals with unhealthy metabolic status. Higher levels of valerate were observed to be associated with an increased risk of cardiovascular disease. Although the mechanism is not fully understood, gut microbiota composition and diversity may link the production of valerate to cardiovascular risk [33]. However, the evidence regarding this association is currently limited. Lactate, a waste of anaerobic metabolism and exercising skeletal muscle, was found to be lower in individuals with MHO compared to those with metabolically unhealthy obesity, but it was relatively higher than in those with metabolically healthy normal weight. One possible explanation is that lactate levels may reflect the status of aerobic metabolism and physical fitness. Individuals with MHO seemed to have a higher level of physical activity than those with metabolically unhealthy obesity (1566 vs. 1484 kcal/day). Further study on direct measurement of aerobic metabolism levels would be more informative. Leucine, a branched-chain amino acid, can induce insulin secretion by stimulating pancreatic β cells, improve insulin signaling, and regulate glucagon-like peptide-1 [34]. Elevated circulating leucine has been repeatedly suggested to be associated with insulin resistance and diabetes risk [29, 35–37]. We observed lower leucine levels in individuals with MHO. Alanine is used as a precursor for gluconeogenesis and induces glucagon secretion, leading to hyperglycemia [38]. A meta-analysis suggested that high alanine is associated with a higher risk of diabetes [39]. However, the effects of alanine on the complications of diabetes are complex, as circulating alanine was found to be inversely associated with microvascular disease in individuals with diabetes [40]. Moreover, increasing attention has been recently committed to a metabolite, trimethylamine N-oxide, due to the association with adverse cardiovascular outcomes [41]. Trimethylamine N-oxide is metabolized from trimethylamine, generated by the gut bacteria from dietary precursors, such as carnitine from red meat consumption. Despite the experimental evidence and cross-sectional analysis, a large prospective study failed to observe the association between plasma trimethylamine N-oxide with diabetes risk [42]. The trimethylamine levels were observed lower in MHO but higher in metabolic unhealthy obesity. These intangible metabolomic alterations might have profoundly influence on the transition to unhealthy metabolism and subsequently change long-term outcomes.

Previous studies in metabolomics have demonstrated that individuals with MHO may exhibit undesirable alterations in their metabolism, regardless of the discrepancy between studies in the definition of MHO, the scope of investigated metabolites, statistical methods, and the clinical profiles of participants [6–9]. For instance, compared to metabolically unhealthy obese individuals or those with normal weight, people with MHO tend to have an intermediated atherogenic lipoprotein profile characterized by elevated levels of VLDL and LDL and reduced levels of HDL [6]. Several case-control studies have also reported similarities between the amino acid patterns of MHO and metabolically unhealthy obesity, including increased levels of alanine and leucine, compared with metabolically healthy normal weight [7, 8]. However, findings on the metabolomic differences between MHO and metabolically unhealthy obesity have been inconsistent [7–9]. Nonetheless, other metabolomics studies have illustrated the adverse impact of obesity on metabolism and outcomes [43, 44]. A metabolomic study identified 49 metabolites that were associated with BMI and contributed to cardiometabolic risks, including glucose, branched-chain and aromatic amino acids, and phospholipids [43]. A large multi-center study involving 7663 individuals used 108 metabolites to develop a metabolomic pattern that could predict BMI and obesity and had prognostic value for type 2 diabetes and mortality [44]. The metabolites contributing to the predictive model were mainly amino acids (such as glutamate, valerate, leucine, glutamine, and valine) and nucleotides, providing insight into the heterogeneity of obesity [44]. Based on the present study and the previous findings, combining metabolites and clinical criteria may benefit the characterization of MHO and improve cardiovascular risk stratification among individuals with obesity.

### Strengths and limitations

The strengths of this present study included the prospective study design and relatively long-term follow-up, well-characterized population-based cohorts from two Europe centers, a large-scale investigation of metabolomics at baseline to illustrate intangible metabolic alterations underlying metabolic health phenotypes, and the inclusion of covariates to eliminate the potential confounding. This study has several limitations. First, the course of metabolic health status was not thoroughly investigated because the second classification did not perform due to lack of necessary information, such as waist-to-hip ratios during the follow-up. Second, the metabolomic profiling focused on small metabolites and did not measure the large metabolites, such as different fatty acids to reflect oxidation and saturated structure. Moreover, the coverage of metabolites was limited in terms of the entire metabolomics of thousands of metabolites involved in systematic metabolism [17]. There is a need for future studies to use more comprehensive metabolomics approaches to fully understand the complex interplay between metabolism, MHO, and cardiovascular health. Third, NMR spectroscopy can quantitatively capture a comprehensive metabolomic pattern for blood samples. The metabolites measured by NMR are highly correlated with the results from clinical chemistry assays, and the association of metabolites measured by different platforms with disease outcomes was consistent [19]. However, the complexity of the samples, such as molecular binding, and the concentration of metabolites can cause overlapped signals [16, 17]. For example, the correlation between the NMR-measured glucose and clinically measured glucose was 0.37. This observed discrepancy could be due to several factors, including spectral crowding and overlap causing multiple glucose signals to be measured as glucose and glucose-composited metabolites, as well as variations in sample preparation and measurement procedures. To validate the reproducibility of metabolite identification and qualification, we applied a high-field NMR spectrometry operating at field strengths of ≥ 600 MHz and used reference compounds and spectral deconvolution. Additionally, we plan to perform additional validation studies in future research, such as high-resolution mass spectrometric techniques and developing advanced spectral fitting algorithms, to better understand the source of variation and improve the accuracy of NMR measurements. Last, new definition was only validated in European populations, and caution should be given when generalize to other populations.

## Conclusions

Individuals classified as MHO seemingly are not at increased cardiovascular risk in a time frame of 10 years. However, the undesirable metabolomic changes associated with cardiovascular risk was more prominent in individual with obesity, irrespective of metabolic health status, suggesting that MHO may requires interventions before the adverse outcome set in. Future experimental studies and clinical trials are needed to improve the biological understanding of our findings and to prove the translational benefits of metabolomic screening in otherwise healthy obese individuals.

## Electronic supplementary material

Below is the link to the electronic supplementary material.

Supplementary Material 1

## Data Availability

The datasets analyzed during the current study are not publicly available due to participants’ privacy protection but are available from the corresponding authors on reasonable request.

## List of abbreviations

BMI: Body mass index
Mets: Metabolic syndrome
MHO: Metabolically healthy obesity
NMR: Nuclear magnetic resonance.
