# Supplementary material for: Cardiovascular risk of metabolically healthy obesity in two european populations: Prevention potential from a metabolomic study
Source: Cardiovasc Diabetol. 2023 Apr 7;22:82. doi: 10.1186/s12933-023-01815-6 (PMC10082537; doi:10.1186/s12933-023-01815-6)
Supplement: Supplementary file 1 — Supplementary Material 1 [file 12933_2023_1815_MOESM1_ESM.docx]

Supplements

***Cardiovascular Risk of Metabolically Healthy Obesity in Two European Populations: Prevention Potential from a Metabolomic Study***

*Dongmei Wei; Vannina González Marrachelli, Jesus D. Melgarejo; Chia-Te Liao; Angie Hu; Stefan Janssens; Peter Verhamme; Lucas Van Aelst; Thomas Vanassche; Josep Redon; Maria Tellez-Plaza; Juan C Martin-Escudero; Daniel Monleon*†*; Zhen-Yu Zhang*†

**Contents**

**Supplemental method:** Metabolomic Profiling **p2**

**Table S1.** Baseline Characteristics of Participants in Two Centers **p3**

**Table S2**. Sensitivity Analysis of Association between Metabolic Phenotypes and Cardiovascular Events **P4**

**Table S3**. Constructed Metabolites-based Factors by Factor Analysis **p5**

**Table S4**. Association of Other Metabolites-based Factors with Cardiovascular Events **p6**

**Figure S1**. The distribution of measured circulating amino acids **p7**

**Figure S2**. The distribution of measured circulating lipids and carbohydrates **p8**

**Figure S3**. The distribution of measured circulating organ acids and other metabolites **p9**

**Figure S4**. Metabolite difference in six subgroups **p10**

**Figure S5**. Significantly different metabolites in metabolically healthy obesity **p12**

**Figure S6**. Standardized Factor Loadings of 32 Significant Metabolites **P13**

**Figure S7**. Correlation between clinically measured glucose and glucose-related metabolites **P14**

**Figure S8**. Cardiovascular Risk-associated Metabolomic Factor in Two Cohorts **P15**

# Metabolomic Profiling

Venous blood samples were centrifuged after collection and supernatant plasma was stored under -80 °C until further analysis. A standard 1-dimensional pulse sequence with water suppression (Bruker Avance 600 spectrometer operating at 600.13 MHz with a 5-mm 1H/13C/15N TXI probe) was used to acquire 1H NMR spectra. A spectral width of 14 ppm and a recycle delay of 1 second were configured to collect 256 free induction decay into 64 k data points. The spectral chemical shift of all spectra was indicated by using the alanine CH3 doublet signal at 1.475 ppm as the reference. Resonances in these spectral regions were assigned according to the Human Metabolome Database and selected two-dimensional NMR spectra.^1^ The metabolite relative abundances were calculated by spectral region area integration and normalization to the total aliphatic spectral area. Topspin 3 1.3 (Bruker Biospin GmbH, Karlsruhe, Germany) and MATLAB® (MathWorks Inc., version 4 2013a) were used for spectra processing and analysis. Two metabolites were jointly reported if a spectral region was contributed by two peaks. The reproducibility of the NMR measurements was assessed by splitting the complete aliphatic spectral region into 0.005 ppm buckets to cancel random noise and maximise the signal-to-noise ratio. The mean bucket difference for all aliphatic spectral regions was 5.1% with a maximum of 7.0% for the spectral bucket having the high-density lipoprotein apolipoprotein signal.

# Table S1.

**Baseline Characteristics of Participants in Two Centers**

| **Characteristics** | **All**  **(n=2339)** | **FLEMENGHO**  **(n=1331)** | **Hortega**  **(n=1008)** |
| --- | --- | --- | --- |
| Number with characteristic (%) |  |  |  |
| Female | 1161 (49.6) | 669 (50.3) | 492 (48.8) |
| Current Smoking | 484 (20.7) | 228 (17.1) | 256 (25.4) |
| Current alcohol assumption | 1457 (62.3) | 936 (70.3) | 521 (51.7) |
| History of CVD | 112 (4.8) | 112 (8.4) | 0 (0.0) |
| Diabetes mellitus | 116 (5.0) | 56 (4.2) | 60 (6.0) |
| Hypertension | 940 (40.2) | 655 (49.2) | 365 (36.2) |
| Treatment of hypertension | 481 (20.6) | 330 (24.8) | 151 (15.0) |
| Lipid-lowering drugs | 252 (10.8) | 199 (15.0) | 53 (5.3) |
| Mean or median of characteristic | | | |
| Age, years | 51.2 ± 16.9 | 51.3 ± 15.9 | 51.1 ± 18.2 |
| Body mass index, kg/m^2^ | 26.4 ± 4.4 | 26.5 ± 4.5 | 26.3 ± 4.2 |
| Waist-to-hip ratio | 0.87 ± 0.09 | 0.88 ± 0.08 | 0.87 ± 0.09 |
| Waist circumference, cm | 90.0 ± 12.8 | 91.0 ± 12.8 | 88.8 ± 12.8 |
| Hip circumference, cm | 102.8 ± 8.7 | 103.4 ± 8.7 | 102.0 ± 8.7 |
| Physical activity, calorie | 1444.8 (977.1-2022.9) | 1710.9 (1332.5-2115.4) | 865.7 (529.2-1491.0) |
| Systolic blood pressure, mmHg | 130.0 ± 18.7 | 130.4 ± 17.3 | 129.6 ± 20.5 |
| Diastolic blood pressure, mmHg | 80.2 ± 10.1 | 80.8 ± 9.7 | 79.3 ± 10.5 |
| Total cholesterol, mmol/L | 5.31 ± 0.98 | 5.07 ± 0.95 | 5.62 ± 0.94 |
| LDL-cholesterol, mmol/L | 3.27 ± 0.88 | 3.04 ± 0.83 | 3.58 ± 0.86 |
| HDL-cholesterol, mmol/L | 1.43 ± 0.37 | 1.46 ± 0.39 | 1.39 ± 0.36 |
| Triglycerides, mmol/L | 1.08 (0.74-1.64) | 1.05 (0.78-1.50) | 1.14 (0.64-1.78) |
| Blood glucose, mmol/L | 4.78 (4.50-5.11) | 4.72 (4.44-5.05) | 5.00 (4.61-5.28) |
| Serum creatinine, mg/dL | 0.86 (0.74-0.99) | 0.89 (0.77-1.01) | 0.82 (0.70-0.97) |
| eGFR, ml/min/1.73m^2^ | 88.7 ± 21.7 | 87.5 ± 18.2 | 90.3 ± 25.5 |

CVD, cardiovascular disease; eGFR, estimated glomerular filtration rate.

# Table S2.

**Sensitivity Analysis of Association between Metabolic Phenotypes and Cardiovascular Events**

|  | **Cardiovascular events:**  **Excluding BMI < 18.5 or ≥40 kg/m^2^** | |  | | **Cardiovascular events:**  **Excluding lipid lowering drugs** | | |  |
| --- | --- | --- | --- | --- | --- | --- | --- | --- |
|  | **HR (95% CI)** | ***P*** | |  | | **HR (95% CI)** | ***P*** | |
| New definition | | | | | | | | |
| MHNW | 1 (reference) |  | |  | | 1 (reference) |  | |
| MHOW | 1.21 (0.57-2.59) | 0.62 | |  | | 1.42 (0.63-3.21) | 0.40 | |
| MHO | 1.10 (0.35-3.43) | 0.87 | |  | | 1.05 (0.29-3.83) | 0.94 | |
| MUHNW | 3.26 (1.71-6.21) | 0.0003 | |  | | 3.52 (1.74-7.13) | 0.0005 | |
| MUHOW | 2.50 (1.34-4.66) | 0.004 | |  | | 2.67 (1.35-5.27) | 0.005 | |
| MUHO | 3.42 (1.81-6.45) | 0.0002 | |  | | 3.57 (1.79-7.15) | 0.0003 | |
| MetS-based definition | | | | | | | | |
| MHNW | 1 (reference) |  | |  | | 1 (reference) |  | |
| MHOW | 0.85 (0.58-1.24) | 0.39 | |  | | 0.84 (0.57-1.23) | 0.36 | |
| MHO | 1.17 (0.73-1.87) | 0.52 | |  | | 1.11 (0.69-1.80) | 0.67 | |
| MUHNW | 2.13 (1.20-3.80) | 0.010 | |  | | 1.87 (0.87-4.05) | 0.11 | |
| MUHOW | 1.60 (1.05-2.43) | 0.027 | |  | | 1.70 (1.07-2.72) | 0.026 | |
| MUHO | 1.81 (1.18-2.78) | 0.006 | |  | | 1.73 (1.09-2.76) | 0.021 | |

HRs were calculated for every standard deviation increment in metabolites and adjusted for the cohort indicator, sex, age, total physical activity, smoking, and alcohol consumption. MHNW, metabolically healthy normal weight; MHOW, metabolically healthy overweight; MHO, metabolically healthy obesity; MUHNW, metabolically unhealthy normal weight; MUHOW, metabolically unhealthy overweight; MUHO, metabolically unhealthy obesity; HR, hazard ratio.

# Table S3.

**Constructed Metabolites-based Factors by Factor Analysis**

|  | **Loading** | **Variance** | **Cumulative Variance** |
| --- | --- | --- | --- |
| Factor 1 | 8.515 | 0.224 | 0.224 |
| Factor 2 | 5.869 | 0.154 | 0.379 |
| Factor 3 | 4.677 | 0.123 | 0.502 |
| Factor 4 | 4.396 | 0.116 | 0.617 |
| Factor 5 | 4.342 | 0.114 | 0.732 |

# Table S4.

**Association of Other Metabolites-based Factors with Cardiovascular Events**

|  | **Cardiovascular Events** | |
| --- | --- | --- |
|  | **HR (95% CI)** | ***P*** |
| All participants* |  |  |
| Factor 1 | 0.96 (0.84-1.11) | 0.61 |
| Factor 3 | 0.88 (0.77-1.01) | 0.064 |
| Factor 4 | 1.04 (0.90-1.20) | 0.62 |
| Factor 5 | 0.89 (0.79-1.01) | 0.075 |
| FLEMENGHO |  |  |
| Factor 1 | 0.91 (0.77-1.07) | 0.24 |
| Factor 3 | 0.91 (0.77-1.07) | 0.25 |
| Factor 4 | 0.88 (0.73-1.06) | 0.18 |
| Factor 5 | 0.90 (0.76-1.06) | 0.19 |
| Hortega |  |  |
| Factor 1 | 1.07 (1.05-0.83) | 0.66 |
| Factor 3 | 1.07 (0.90-0.72) | 0.39 |
| Factor 4 | 1.06 (1.28-0.99) | 0.061 |
| Factor 5 | 1.06 (1.01-0.80) | 0.95 |

HRs were adjusted for sex, age, total physical activity, smoking, and alcohol consumption. *HRs for all participants were additionally adjusted for the cohort indicator.


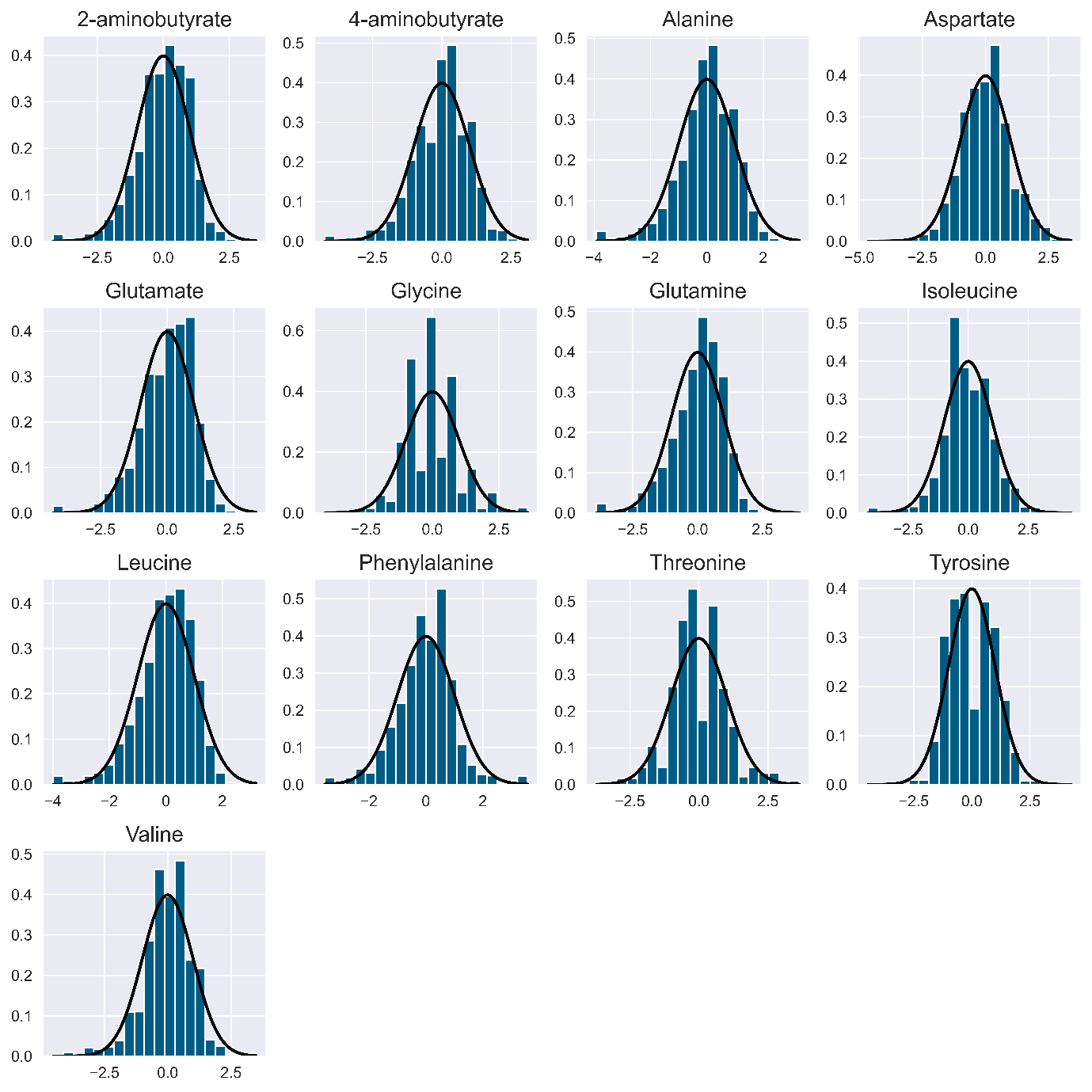


# Figure S1.

The distribution of measured circulating amino acids after data transformation and normalization in 2218 participants from the general population.


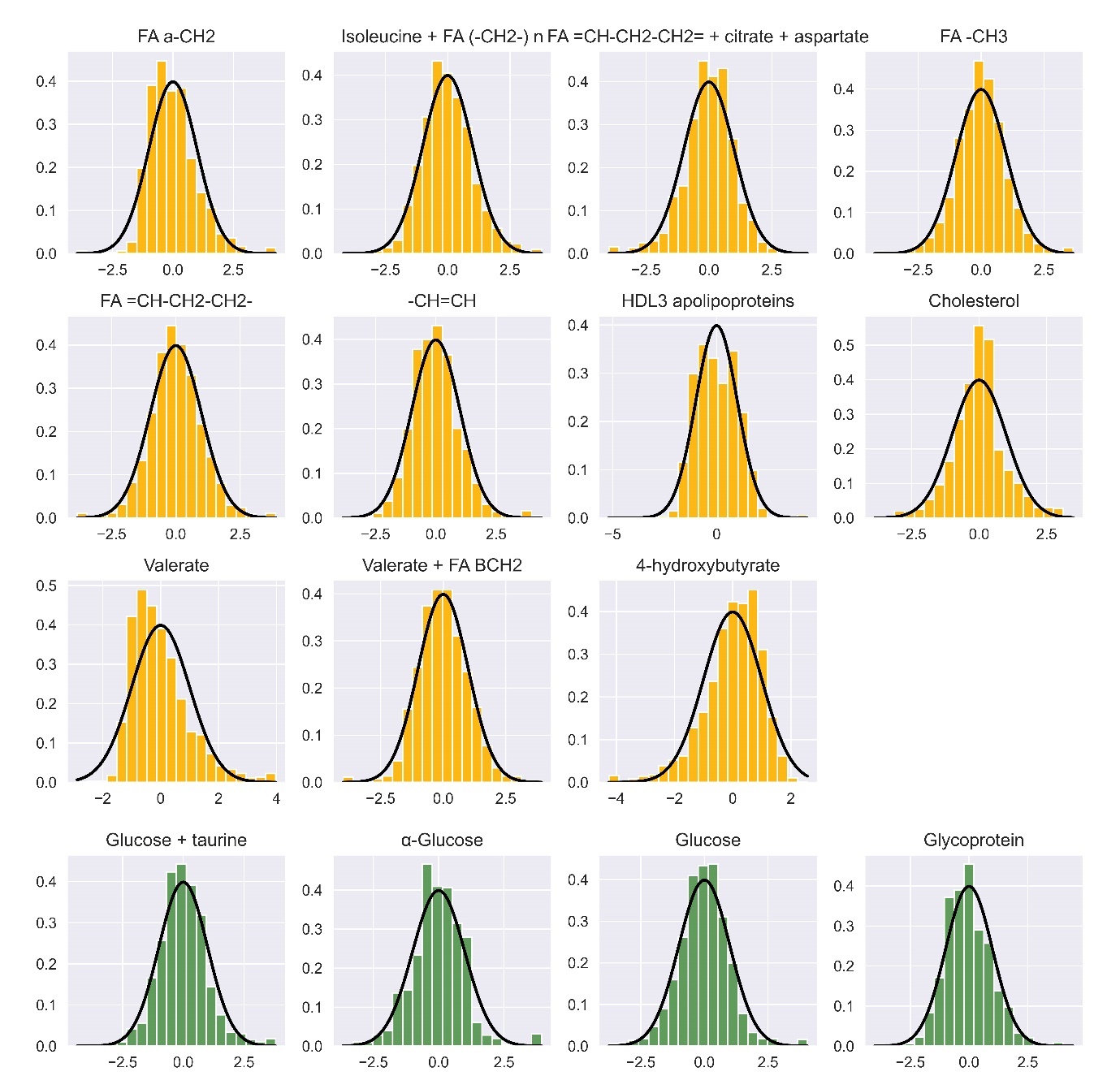


# Figure S2.

The distribution of measured circulating lipids and carbohydrates after data transformation and normalization in 2218 participants from the general population.


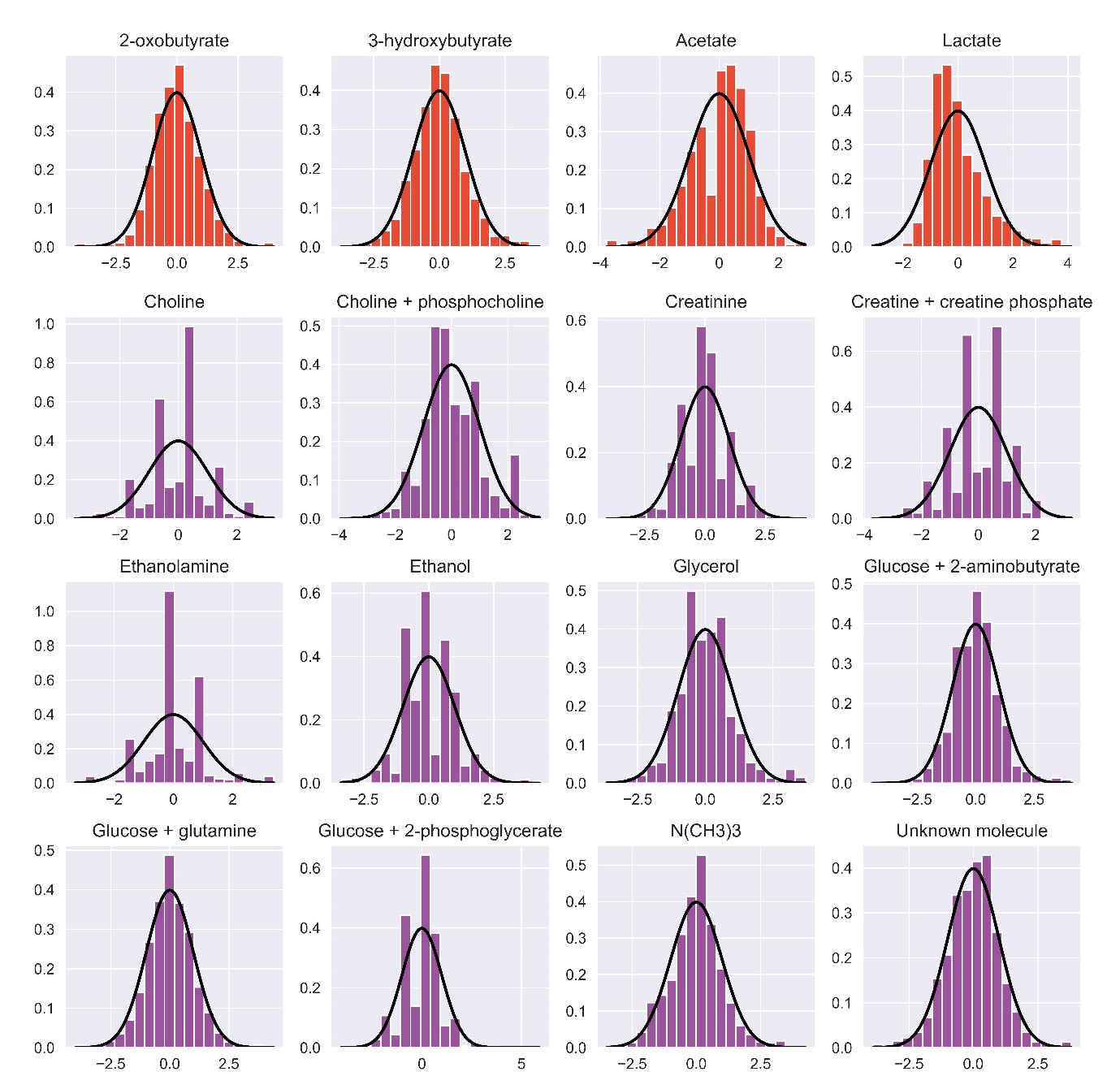


# Figure S3.

The distribution of measured circulating organ acids and other metabolites after data transformation and normalization in 2218 participants from the general population.


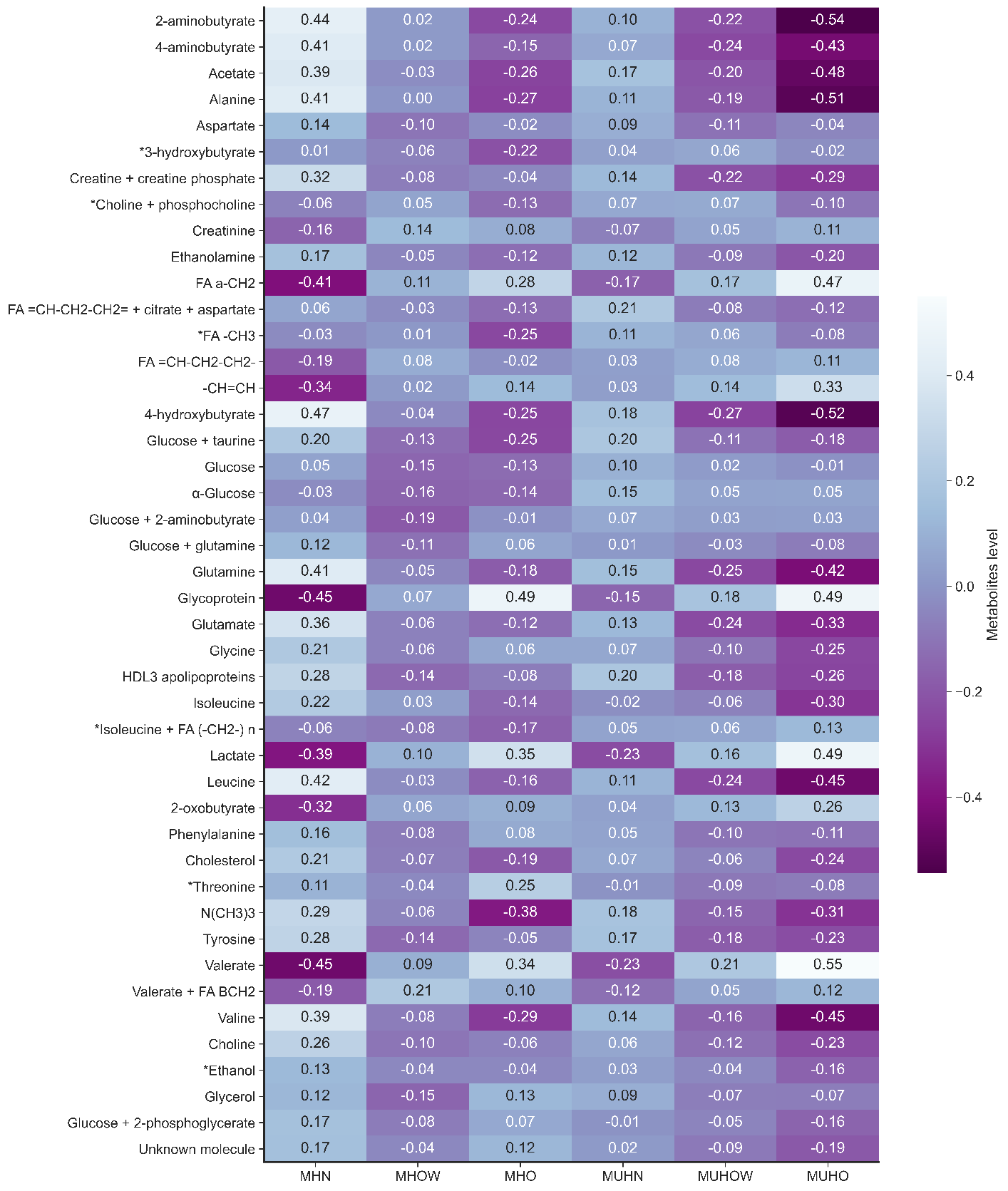


# Figure S4.

**Metabolite difference in six subgroups.** The number in each cell represents the mean Z score of a metabolite in each subgroup. The large Z score denotes a higher level of metabolites. There were 38 metabolites significantly different in group-wise comparison, except for 6 metabolites marked with a star (*) in the figure. The measured metabolites were unitless and expressed as relative levels because the spectral vector was normalized to the total spectral area excluding residual water signals to minimize the effects of variable dilution of the sample. After scaling and normalization in data processing, the metabolite level was expressed as Z scores. Significant metabolites were detected by group-wise comparison (the Kruskal-Wallis test) and their P values were corrected by the Bonferroni correction.


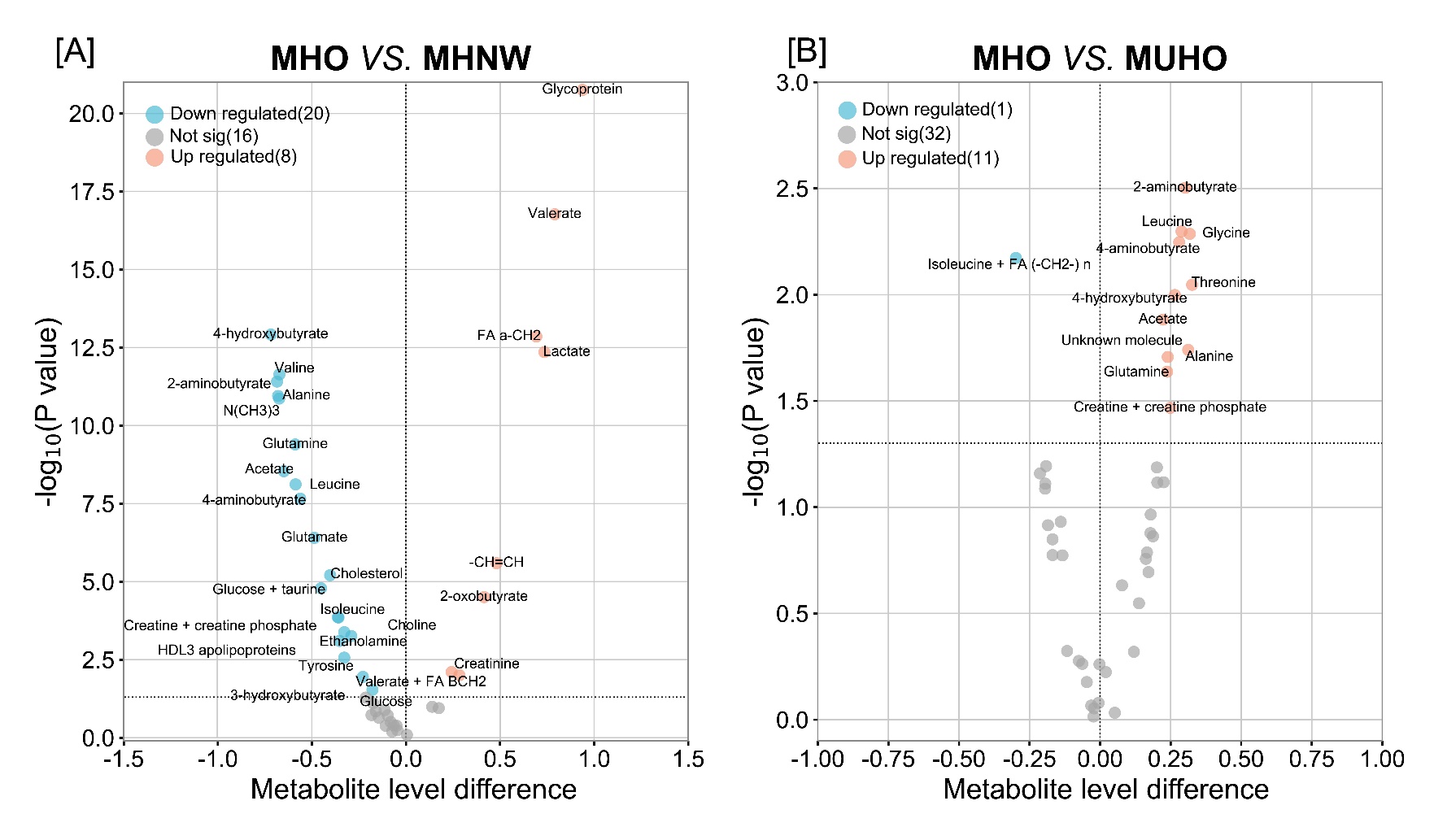


# Figure S5.

**Significantly different metabolites in metabolically healthy obesity (MHO) compared to two metabolic phenotypes. A.** Compared with metabolically healthy normal weight (MHNW), 28 metabolites were significantly different in MHO, including 20 downregulated and 8 upregulated metabolites. **B**. 12 metabolites significantly differed in individuals with MHO compared to metabolically healthy obesity (MUHO). The metabolite level difference denotes the mean difference in Z scores in each metabolite.


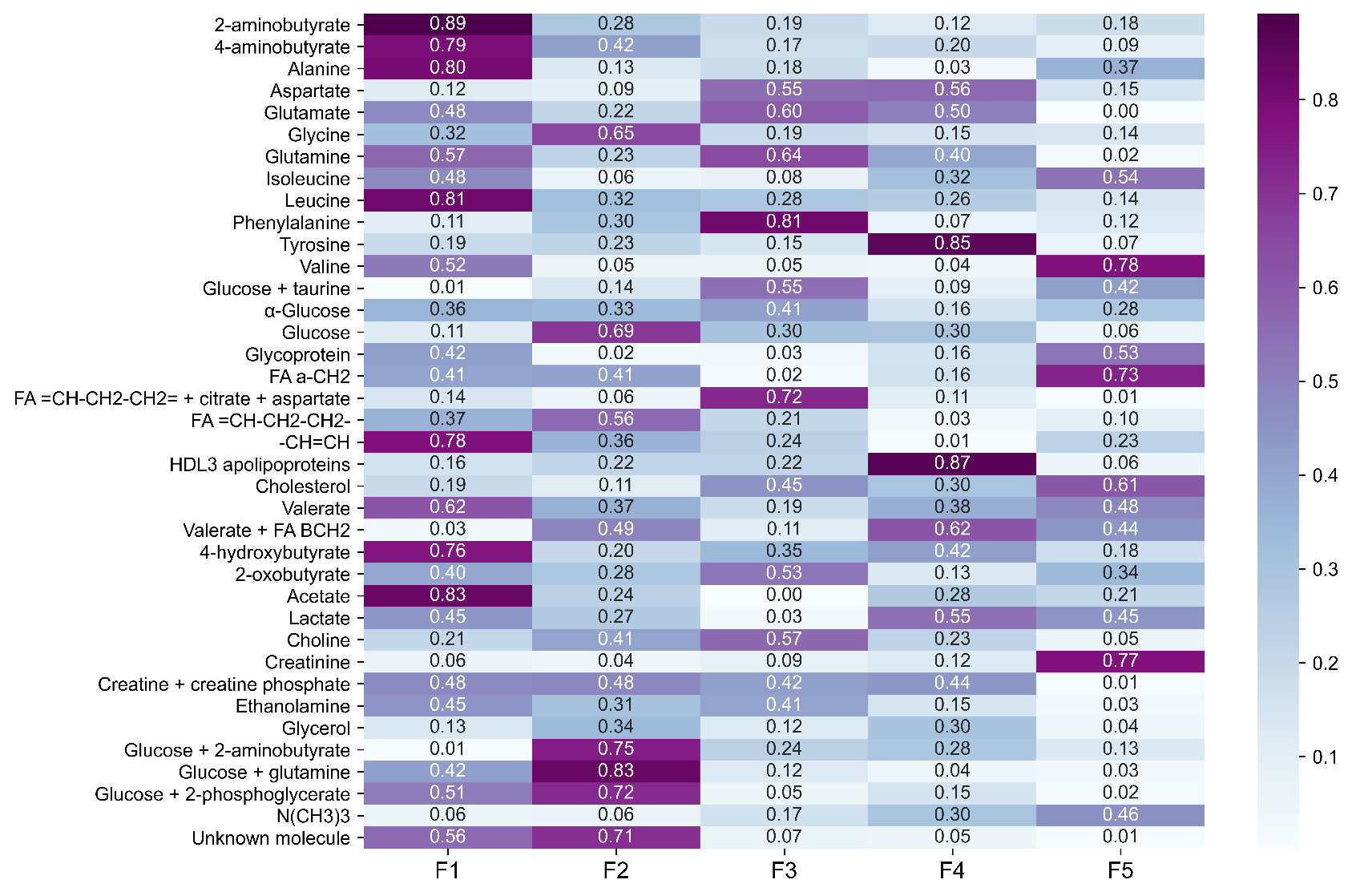


# Figure S6.

**Standardized Factor Loadings of 38 Significant Metabolites.** Factor loadings denote the correlation between metabolites and factors. A factor loading score of > 0.6 is commonly considered practical significance.


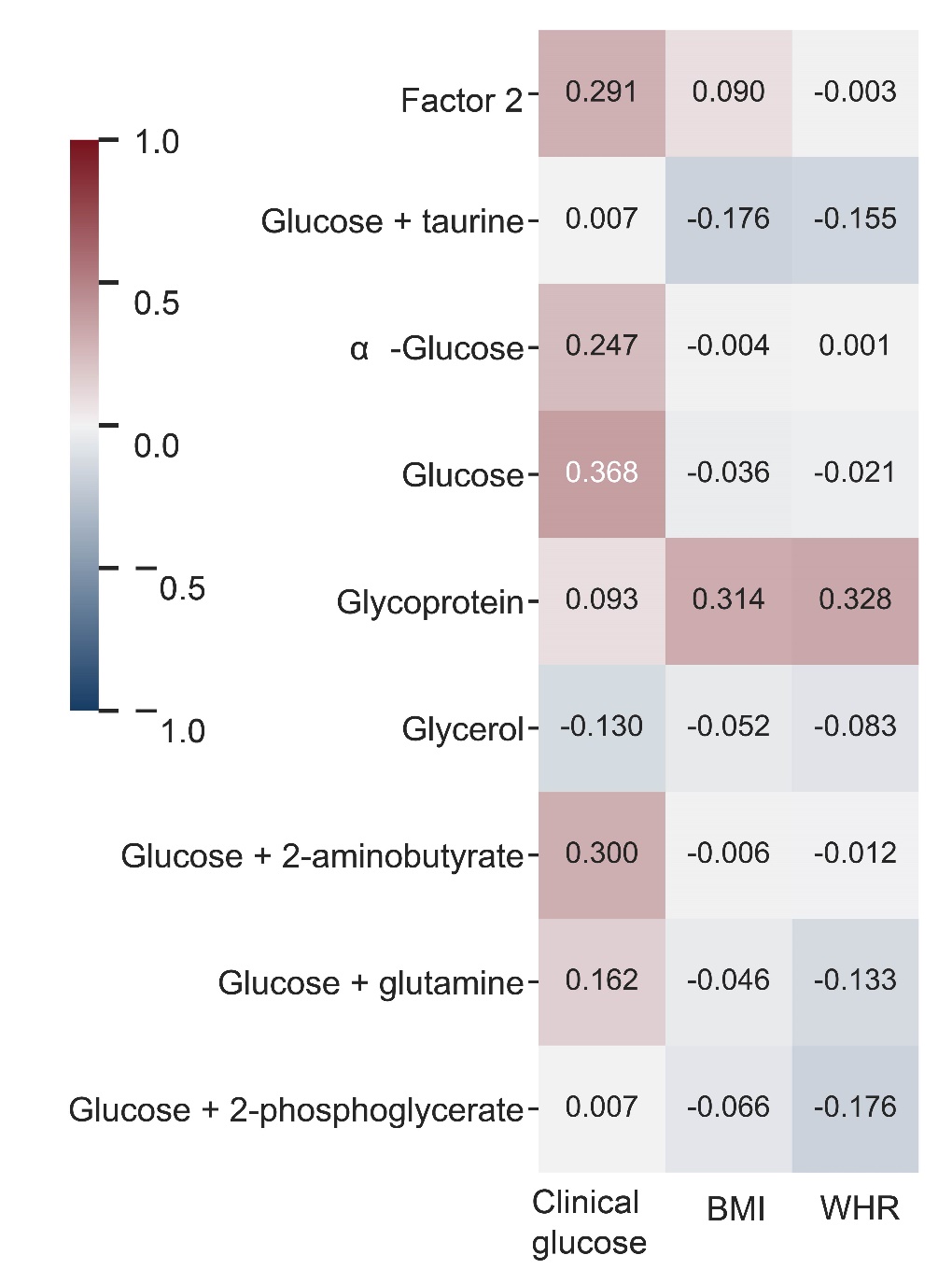


# Figure S7.

**Correlation between clinically measured glucose and glucose-related metabolites.** Clinically measured glucose and glucose-related metabolites had a low-to-moderate correlation. The number denotes the correlation coefficients. BMI, body mass index; WHR, waist-to-hip ratio.


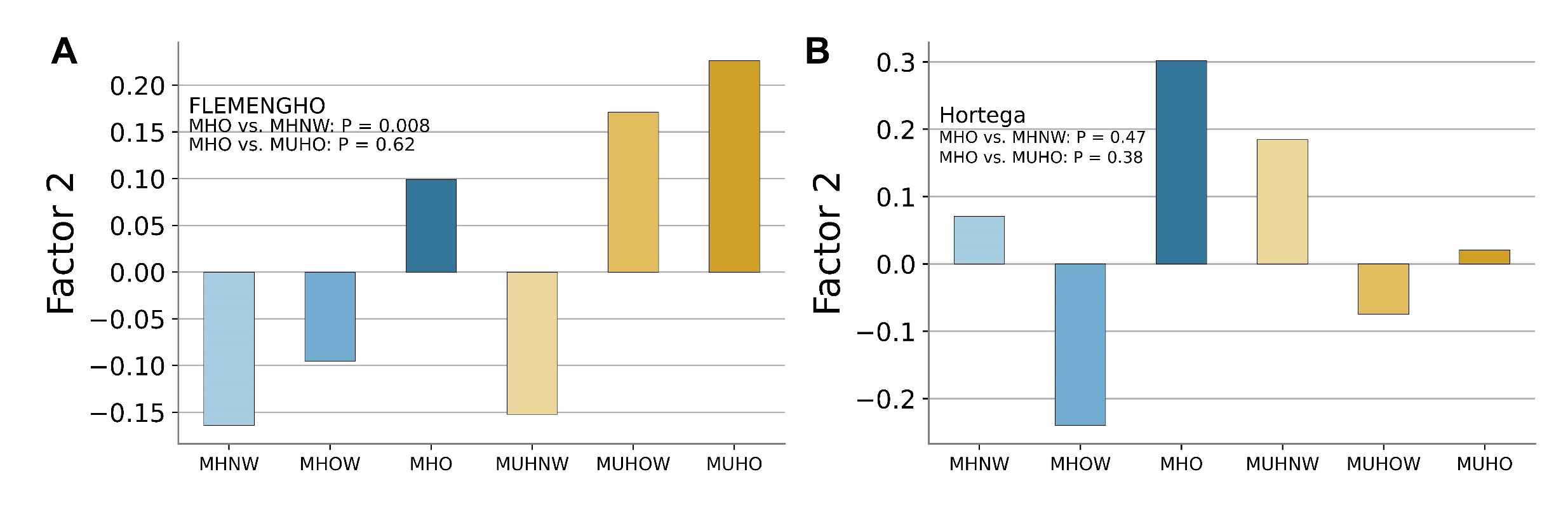


# Figure S8.

Cardiovascular Risk-associated Metabolomic Factor in **Two Cohorts.** MHNW, metabolically healthy normal weight; MHOW, metabolically healthy overweight; MHO, metabolically healthy obesity; MUHNW, metabolically unhealthy normal weight; MUHOW, metabolically unhealthy overweight; MUHO, metabolically unhealthy obesity.
